# The SmWRKY32-SmbHLH65/SmbHLH85 regulatory module mediates tanshinone biosynthesis in *Salvia miltiorrhiza*

**DOI:** 10.1093/hr/uhaf096

**Published:** 2025-03-25

**Authors:** Xiumin Nie, Xueying Li, Bingbing Lv, Shuai Shao, Bin Zhang, Juane Dong

**Affiliations:** College of Life Sciences, Northwest A&F University, No. 22 Xinong Road, Yangling, Shaanxi, 712100, China; College of Life Sciences, Northwest A&F University, No. 22 Xinong Road, Yangling, Shaanxi, 712100, China; College of Life Sciences, Northwest A&F University, No. 22 Xinong Road, Yangling, Shaanxi, 712100, China; College of Life Sciences, Northwest A&F University, No. 22 Xinong Road, Yangling, Shaanxi, 712100, China; College of Life Sciences, Northwest A&F University, No. 22 Xinong Road, Yangling, Shaanxi, 712100, China; College of Life Sciences, Northwest A&F University, No. 22 Xinong Road, Yangling, Shaanxi, 712100, China

## Abstract

Tanshinones are valuable compounds found in *Salvia miltiorrhiza*, and gaining a deeper understanding of their transcriptional regulation mechanisms is a key strategy for increasing their content. Previous research revealed that SmWRKY32 acts as a repressor of tanshinone synthesis. This study identified the SmbHLH65 transcription factor, whose expression was significantly reduced in the *SmWRKY32* overexpression transcriptome. Overexpression of *SmbHLH65* stimulated tanshinone accumulation, while its silencing resulted in a decrease in tanshinone content. However, SmbHLH65 does not directly target the key enzyme genes involved in tanshinone synthesis. Subsequently, we discovered the SmbHLH65-interacting protein SmbHLH85. SmbHLH85 facilitates tanshinone biosynthesis by directly upregulating *SmDXS2* and *SmCPS1*. Further investigation demonstrated that SmbHLH65 not only promotes the expression of *SmbHLH85* but also enhances its binding to the promoters of *SmDXS2* and *SmCPS1*, thereby amplifying the activation of these biosynthetic genes. Additionally, SmWRKY32 directly binds to the *SmbHLH65* promoter to suppress its activity. In summary, these findings reveal that the regulatory module SmWRKY32-SmbHLH65/SmbHLH85 controls tanshinone synthesis in *S. miltiorrhiza*. This study uncovers a novel transcriptional regulatory mechanism, offering fresh insights into the complex network controlling tanshinone biosynthesis.

## Introduction

*Salvia miltiorrhiza* is a well-known traditional herb used to treat cardiovascular diseases [1, 2]. In *S. miltiorrhiza*, the primary tanshinones include cryptotanshinone (CT), dihydrotanshinone (DT), tanshinone IIA (TIIA), and tanshinone I (TI) [3, 4]. Tanshinones are synthesized through three stages: the production of the universal diterpene precursor geranylgeranyl pyrophosphate (GGPP), the cyclization of GGPP to generate miltiradiene, and the modification of miltiradiene to produce various components of tanshinone [5, 6]. Many key enzyme genes that participated in the tanshinone synthetic pathway have been found and characterized, including 1-deoxy-d-xylulose-5-phosphate synthase (*SmDXS2*) [7], 1-deoxy-d-xylulose-5-phosphate reductoisomerase (*SmDXR*) [8], geranylgeranyl diphosphate synthase (*SmGGPPS1*) [9], copalyl diphosphate synthase (*SmCPS1*) [10], kaurene synthase-like (*SmKSL1*) [11], and various cytochrome P450 enzyme genes (*SmCYP76AH1*, *SmCYP76AH3*, *SmCYP76AK1*, *SmCYP76AK2*, and *SmCYP76AK3*) [12–14]. Overexpression of single or multiple key enzyme genes involved in tanshinone synthesis through transgenic technology can dramatically increase tanshinone content [15, 16].

Transcriptional regulation is crucial for controlling tanshinone biosynthesis, and numerous transcription factors have been identified. For instance, SmGRAS3 promotes tanshinone biosynthesis via stimulating *SmKSL1* transcription [17]. SmMYB97 increases tanshinone content by upregulating *SmCPS1* and *SmKSL1* [18]. Similarly, overexpression of *SmbHLH148* also enhances tanshinone biosynthesis [19]. The WRKY protein is a crucial regulator of tanshinone synthesis in *S. miltiorrhiza*. For example, SmWRKY1 and SmWRKY2 improve tanshinone accumulation by activating *SmDXR* and *SmCPS1*, respectively [20, 21]. Additionally, SmWRKY61 positively regulates tanshinone biosynthesis [22]. In contrast, SmWRKY32 decreases tanshinone yield by downregulating the transcript levels of *SmGGPPS1*, *SmCPS1*, and *SmERF128* [23]. Moreover, SmWRKY34 negatively affects tanshinone biosynthesis by reducing the expression of *SmGGPPS1* and *SmbZIP3* [24]. However, the transcriptional complexes that regulate tanshinone biosynthesis remain largely unknown.

The basic helix–loop–helix (bHLH) protein comprises a conserved bHLH domain consisting of approximately 50–60 amino acids. The N-terminal possesses a basic region that is roughly 10–15 residues long and is rich in basic amino acids, which recognize and bind to the G-box (CACGTG) or E-box (CANNTG) in the target gene promoter. The C-terminal HLH region comprises approximately 40 amino acids and contains two α-helices and a variable loop [25, 26]. The HLH region facilitates dimer formation between bHLH proteins [27–29]. In brief, bHLH proteins can identify and combine with G-box and E-box elements in downstream target gene promoters and interact with other transcription factors to cooperatively activate or inhibit gene expression.

bHLH transcription factors have been shown to influence plant developmental processes, abiotic stress responses, as well as the production of secondary metabolites [29–31]. For example, MdbHLH3 directly regulates auxin signaling to control apple leaf shape [32]. The bHLH transcription factor OsPIL15 positively regulates grain size by elevating the expression of *OsPUP7* in rice [33]. NtbHLH123 contributes to salt stress by regulating the transcription of *NtRbohE* and ROS production in tobacco [34]. SlbHLH96 positively regulates drought stress in tomato [35]. In tomato, the bHLH transcription factor SlJAF13 raises anthocyanin biosynthesis [36]. Overexpression of *TaMYC2* enhances triterpene biosynthesis in *Taraxacum antungense* Kitag [37]. In addition, bHLH transcription factors can also form dimers to exert their effects [38]. AtbHLH121 interacts with AtbHLH34, AtbHLH104, AtbHLH105, and AtbHLH115 to modulate Fe homeostasis in *Arabidopsis thaliana* [39]. Heterodimers formed between SmMYC2 and SmbHLH37 or SmbHLH60 antagonistically control phenolic acid synthesis in *S. miltiorrhiza* [40, 41]. However, bHLH heterodimers involved in regulating tanshinone synthesis have not yet been reported. Therefore, studying the bHLH–bHLH complex could expand understanding of the mechanism by which the bHLH family controls secondary metabolite synthesis in *S. miltiorrhiza* and offer insights for promoting the accumulation of these metabolites.

This study identified the SmbHLH65 transcription factor from the overexpression transcriptome of *SmWRKY32*. Overexpression of *SmbHLH65* significantly increases tanshinone content, while RNA interference markedly reduces tanshinone yield. However, SmbHLH65 does not directly target a specific gene but functions through its interaction with SmbHLH85. SmbHLH85 promotes tanshinone biosynthesis by stimulating *SmDXS2* and *SmCPS1* transcription. Additionally, SmbHLH65 positively regulates *SmbHLH85* expression and strengthens its transcriptional activation of target genes. Furthermore, SmWRKY32 directly binds to the *SmbHLH65* promoter, repressing its expression. In summary, this study reveals a new module, SmWRKY32-SmbHLH65/SmbHLH85, involved in tanshinone synthesis, further enriching the transcriptional network that modulates tanshinone production.

**Figure 1:**
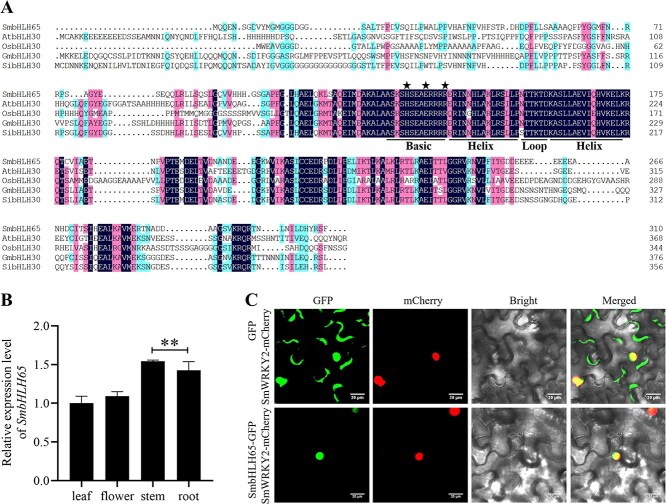
Molecular characterization and subcellular localization of SmbHLH65. (A) Multiple sequence alignment of SmbHLH65 with other plant species bHLH proteins, including AtbHLH30 (AT1G68810.1), OsbHLH30 (XP_015648851.1), GmbHLH30 (XP_006573268.1), and SibHLH30 (XP_011077316.1). The bHLH domain is indicated by lines, and the HER motif (His5-Glu9-Arg13) is marked by a pentagram. (B) The *SmbHLH65* relative expression levels in leaf, flower, stem, and root tissues of *S. miltiorrhiza.* Leaf samples served as the experimental control. Error bars represent standard deviation (SD) (Student’s *t*-test, ^**^P < 0.01). (C) Subcellular localization of SmbHLH65 in tobacco. SmbHLH65-GFP was cotransformed with the nuclear marker SmWRKY2-mCherry. Scale bar = 20 μm.

## Results

### Cloning and characterization of *SmbHLH65*

A total of 127 bHLH members were identified in *S. miltiorrhiza* and categorized into 25 subgroups (A to Y). SmbHLH65 belonged to subgroup E [42]. *SmbHLH65* has a 933-bp full-length open reading frame (ORF) encoding 310 amino acids. A multiple sequence alignment of SmbHLH65 with related bHLH proteins from *Arabidopsis*, *Oryza sativa*, *Glycine max*, and *Sesamum indicum* showed that SmbHLH65 contained a highly conserved bHLH domain (Fig. 1A). Tissue expression analysis revealed that *SmbHLH65* was preferentially expressed in stem and root (Fig. 1B). Subcellular localization of SmbHLH65 was examined in *Nicotiana benthamiana* leaves. The experiment showed that the SmbHLH65 signal was present in the nuclei (Fig. 1C).

### SmbHLH65 promotes tanshinone accumulation

**Figure 2:**
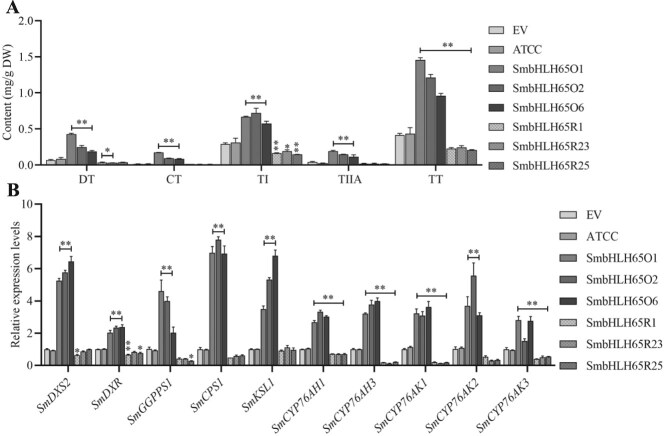
SmbHLH65 promotes tanshinone biosynthesis by upregulating key biosynthetic genes. (A) Content of tanshinones in EV, ATCC, and *SmbHLH65* transgenic hairy roots. DW refers to dry weight. DT, CT, TI, and TIIA. TT was the sum of these four types of tanshinones. (B) Transcription levels of key enzyme genes associated with tanshinone biosynthesis in *SmbHLH65* transgenic lines. Error bars indicate SD. Ordinary one-way ANOVA with multiple comparisons, ^**^P < 0.01, ^*^P < 0.05.

To ascertain the role of SmbHLH65 in tanshinone synthesis, three independent overexpression (SmbHLH65O1, SmbHLH65O2, and SmbHLH65O6) or RNA interference (SmbHLH65R1, SmbHLH65R23, and SmbHLH65R25) lines were created for further investigation (Fig. S1A). High-performance liquid chromatography (HPLC) was employed to detect the tanshinone content in hairy roots EV, ATCC, and transgenic *SmbHLH65*. Compared with the EV lines, the DT, CT, TI, TIIA, and total tanshinone (TT) content significantly increased in SmbHLH65O lines. On average, DT levels elevated by 4.3 times, CT by 8.2 times, TI by 2.3 times, TIIA by 3.7 times, and TT by 2.9 times. In contrast, the *SmbHLH65R* lines showed an average reduction in DT, TI, and TT accumulation by 51%, 43%, and 45%, respectively, while the levels of CT and TIIA displayed no substantial difference (Fig. 2A). Next, quantitative real-time polymerase chain reaction (qRT-PCR) assays were carried out to analyze the transcription of key enzyme genes associated with tanshinone biosynthesis in EV, ATCC, and transgenic *SmbHLH65* lines. In the SmbHLH65O lines, transcripts of *SmDXS2*, *SmDXR*, *SmGGPPS1*, *SmCPS1*, *SmKSL1*, *SmCYP76AH1*, *SmCYP76AH3*, *SmCYP76AK1*, *SmCYP76AK2*, and *SmCYP76AK3* were substantially upregulated, with average increases of 5.8, 2.3, 3.5, 7.2, 5.2, 3.0, 3.7, 3.3, 4.1, and 2.4 times, respectively. In contrast, the SmbHLH65R lines showed a marked decrease in the expression levels of *SmDXR*, *SmCYP76AH1*, *SmCYP76AH3*, *SmCYP76AK1*, and *SmCYP76AK3* (Fig. 2B). These findings show that SmbHLH65 positively modulates tanshinone biosynthesis. Given the differential expression of tanshinone biosynthesis genes in the EV, ATCC, SmbHLH65O, and SmbHLH65R lines, along with promoter analysis confirming the presence of G/E-box motifs in these enzyme gene promoters, it is suggested that SmbHLH65 may regulate tanshinone biosynthesis by interacting with these motifs. To determine whether SmbHLH65 directly binds to these promoters, yeast one-hybrid (Y1H) experiments were conducted. However, the Y1H results indicated that SmbHLH65 does not directly bind to the promoters of these enzyme genes (Fig. S2). These findings suggest that SmbHLH65 may regulate tanshinone biosynthesis indirectly through other regulatory factors.

### SmbHLH65 interacts with SmbHLH85

**Figure 3:**
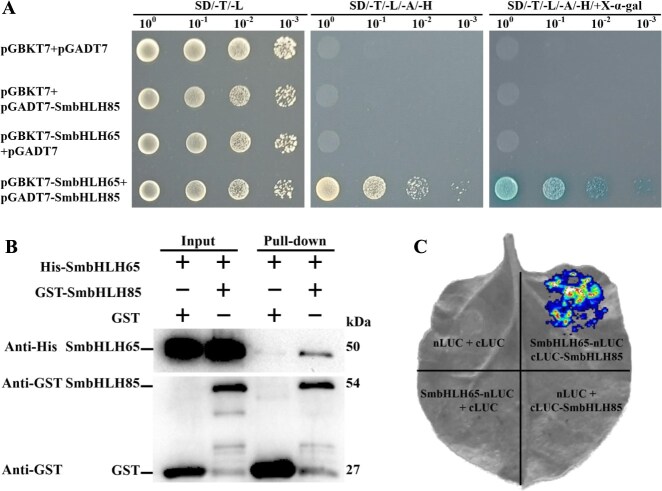
SmbHLH65 interacts with SmbHLH85. (A) Y2H experiments demonstrated the interaction of SmbHLH65 with SmbHLH85. (B) Pull-down assays confirmed the interaction of SmbHLH65 with SmbHLH85. (C) Split-luciferase complementation assays indicated that SmbHLH65 interacts with SmbHLH85 in tobacco leaves.

Since SmbHLH65 cannot directly bind to the promoters of genes involved in tanshinone biosynthesis, we speculated that it may regulate tanshinone accumulation through interactions with other transcription factors. To identify additional transcription factors that may interact with SmbHLH65, SmbHLH65 acted as a bait for yeast two-hybrid (Y2H) screening. Four transcription factors were identified as potential interactors with SmbHLH65. We employed yeast two-hybrid experiments to validate these protein interactions. Among the identified factors, SmAGL14, SmbHLH37, and SmbHLH85 exhibited interactions with SmbHLH65 in yeast, while SmNAC2 did not (Fig. 3A and Fig. S3). The biological function of SmAGL14 has not yet been reported, while AGL14 in *Arabidopsis* is known to be involved in flower development [43, 44]. SmbHLH37 has been identified as a negative regulator of phenolic acid synthesis [41]. SmbHLH85 was chosen for further research. Additionally, pull-down experiments were employed to test whether SmbHLH65 interacts with SmbHLH85. The His-SmbHLH65 fusion protein was successfully captured by GST-SmbHLH85 but not by GST alone (Fig. 3B). Further validation was achieved through luciferase complementation imaging (LCI) assays, where fluorescence signals were exclusively observed at the coinjected sites of SmbHLH65-nLUC and cLUC-SmbHLH85 (Fig. 3C). These results confirm that SmbHLH65 interacts with SmbHLH85.

### Characterization of SmbHLH85

*SmbHLH85* has a coding sequence of 735 bp, encoding 244 amino acids. According to Zhang *et al.* [42], SmbHLH85 was classified into subgroup P. Multiple sequence alignments with related bHLH proteins from *Arabidopsis*, *S. indicum*, *Vitis vinifera*, and *Phtheirospermum japonicum* revealed that SmbHLH85 contained a conserved bHLH domain (Fig. S5A). Tissue expression analysis indicated that *SmbHLH85* was expressed higher in flowers than in the stem, root, and leaf (Fig. S5B). Additionally, subcellular localization studies revealed that SmbHLH85 was specifically localized to the nucleus (Fig. S5C).

### SmbHLH85 positively correlates with tanshinone content

**Figure 4:**
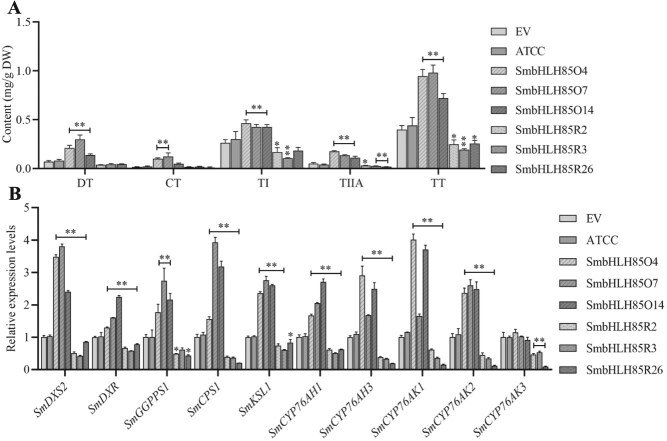
SmbHLH85 actively regulates tanshinone biosynthesis by modulating biosynthetic pathway genes. (A) Tanshinone concentration in overexpressing and silencing *SmbHLH85*. DT, CT, TI, and TIIA. TT was the sum of DT, CT, TI, and TIIA. (B) Transcript levels of key tanshinone synthesis enzyme genes in EV, ATCC, and *SmbHLH85* transgenic lines. Error bars indicate SD. Ordinary one-way ANOVA with multiple comparisons, ^**^P < 0.01, ^*^P < 0.05.

To clarify the biological function of SmbHLH85, transgenics were successfully generated for further analysis. Three high-expression lines (SmbHLH85O4, SmbHLH85O7, and SmbHLH85O14) and three low-expression lines (SmbHLH85R2, SmbHLH85R3, and SmbHLH85R26) were selected for detailed study (Fig. S1B). HPLC assays revealed that the tanshinone components and TT content in the SmbHLH85O lines were markedly elevated compared with the EV. Specifically, DT content increased by 2.0 to 4.2 times, CT by 3.2 to 8.2 times, TI by 1.6 to 1.8 times, TIIA by 2.2 to 3.5 times, and TT by 1.8 to 2.5 times. In contrast, the *SmbHLH85R* lines showed reductions in TI, TIIA, and TT by 31–60%, 42–65%, and 36–51%, respectively, with DT and CT levels remaining largely unchanged (Fig. 4A). qRT-PCR analysis was then performed to examine the transcript levels of key enzyme genes associated with tanshinone biosynthesis in EV, ATCC, and transgenic *SmbHLH85* lines. Compared with the EV, the SmbHLH85O lines displayed dramatically upregulated expression of *SmDXS2*, *SmDXR*, *SmGGPPS1*, *SmCPS1*, *SmKSL1*, *SmCYP76AH1*, *SmCYP76AH3*, *SmCYP76AK1*, and *SmCYP76AK2*. However, no substantial changes were observed in *SmCYP76AK3*. Conversely, the transcript levels of these enzyme genes were markedly reduced in the SmbHLH85R lines (Fig. 4B). These results indicate that SmbHLH85 promotes tanshinone synthesis.

### SmbHLH85 binds *SmDXS2* and *SmCPS1* promoters and activates their transcription

**Figure 5:**
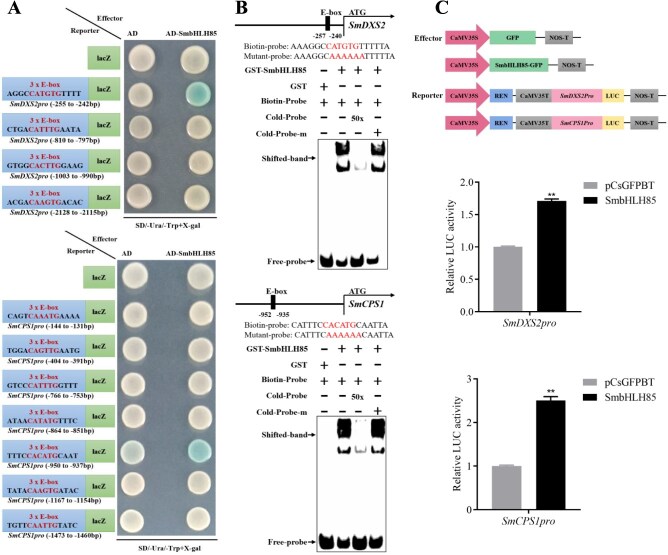
SmbHLH85 activates the transcription of *SmDXS2* and *SmCPS1* through direct binding to their respective promoters. (A) Y1H assays displayed that SmbHLH85 binds to *SmDXS2* and *SmCPS1* promoters. (B) EMSA experiments showed that SmbHLH85 binds to the biotin-labeled probes derived from the promoter of *SmDXS2* and *SmCPS1*. m, mutant. (C) Dual-LUC assays exhibited that SmbHLH85 activates the transcription of both *SmDXS2* and *SmCPS1*. Error bars represent SD (Student's *t*-test, ^**^P < 0.01).

To investigate whether SmbHLH85 directly binds to the promoters of key enzyme genes, Y1H experiments were conducted, revealing that SmbHLH85 specifically binds to the promoters of *SmDXS2* (−255 to −242 bp relative to ATG start codon) and *SmCPS1* (−950 to −937 bp relative to ATG) (Fig. 5A and Fig. S4). This binding was further confirmed by electrophoretic mobility shift assay (EMSA), which demonstrated that SmbHLH85 interacts with the E-box motif in the promoters of *SmDXS2* and *SmCPS1*. In these assays, the GST protein alone showed no binding to the E-box, as indicated by the absence of shifted bands. In contrast, the GST-SmbHLH85 fusion protein bound to the E-box, resulting in the appearance of shifted bands. When a 50-fold excess of unlabeled cold competitive probes was added, the shifted band visibly weakened, whereas the introduction of mutated unlabeled cold probes at the same amount as the biotin-labeled probes had no effect on the shifted band (Fig. 5B). Dual-luciferase (Dual-LUC) assays were then employed to test whether SmbHLH85 modulates *SmDXS2* and *SmCPS1*. The analysis showed that SmbHLH85 dramatically upregulated *SmDXS2* expression by 1.7-fold and *SmCPS1* by 2.5-fold (Fig. 5C). These findings indicate that SmbHLH85 directly binds to the *SmDXS2* and *SmCPS1* promoters and activates their expression.

### SmbHLH65 promotes the transcription of *SmbHLH85* and enhances its transcriptional activation of downstream target genes

**Figure 6:**
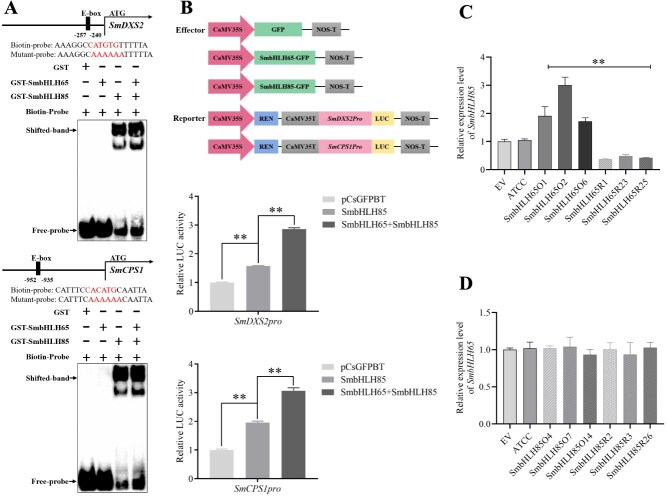
SmbHLH65 positively regulates the expression of *SmbHLH85* and enhances the activation of *SmDXS2* and *SmCPS1* mediated by SmbHLH85. (A) EMSA assays demonstrated that SmbHLH65 strengthens the binding of SmbHLH85 to the *SmDXS2* and *SmCPS1* promoters. (B) Dual-LUC assays indicated that SmbHLH65 amplifies the transcriptional activation of *SmDXS2* and *SmCPS1* by SmbHLH85. (C) Relative expression levels of *SmbHLH85* in *SmbHLH65* overexpression and RNAi lines. (D) Transcription levels of *SmbHLH65* in *SmbHLH85* transgenic hair roots. Error bars represent SD (Student’s *t*-test, ^**^P < 0.01).

To assess whether SmbHLH65 interacts with SmbHLH85 and affects the binding of SmbHLH85 to the *SmDXS2* and *SmCPS1* promoters, EMSA assays were conducted. Results indicated that neither the GST protein nor the GST-SmbHLH65 fusion protein bound to the E-box element, as no shifted bands were observed. However, the GST-SmbHLH85 fusion protein successfully bound to the E-box element, producing a distinct shifted band. Notably, the addition of an equivalent amount of GST-SmbHLH65 protein further intensified the shifted band (Fig. 6A). These findings suggest that SmbHLH65 strengthens the binding of SmbHLH85 to the *SmDXS2* and *SmCPS1* promoters. To explore whether this interaction influences the regulatory activity of SmbHLH85 on *SmDXS2* and *SmCPS1*, Dual-LUC assays were carried out. The results showed that the relative luciferase activity in coexpressing *SmbHLH65*, *SmbHLH85*, and the target gene promoters was dramatically higher compared with coexpressing *SmbHLH85* with the target gene promoters alone (Fig. 6B). This indicates that SmbHLH65 enhances the transcriptional activity of SmbHLH85 on *SmDXS2* and *SmCPS1*. The promoter analysis of *SmbHLH65* and *SmbHLH85* revealed that both contain G/E-box motifs to which bHLH proteins can bind. However, it remains unclear whether a regulatory relationship exists between SmbHLH65 and SmbHLH85. To investigate this potential regulatory relationship, qRT-PCR analysis was performed. As shown in Fig. 6C, the relative expression of *SmbHLH85* was significantly upregulated in the *SmbHLH65* overexpression lines, while it was markedly downregulated in the RNAi lines. Additionally, as illustrated in Fig. 6D, the transcript level of *SmbHLH65* did not exhibit any significant changes in the *SmbHLH85* transgenic hair roots. These results indicate that SmbHLH65 positively regulates the expression of *SmbHLH85*, whereas SmbHLH85 does not modulate the transcript level of *SmbHLH65*.

### *SmbHLH65* is regulated by SmWRKY32

**Figure 7:**
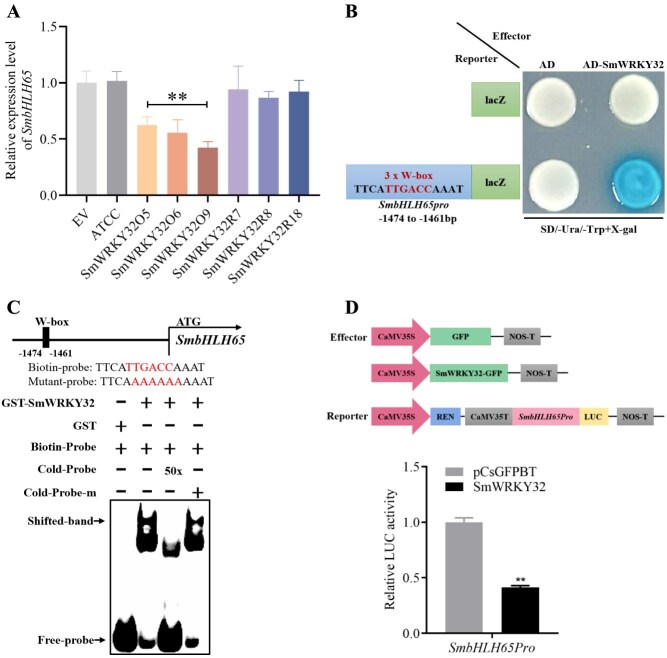
SmWRKY32 binds to the *SmbHLH65* promoter and suppresses its transcription activity. (A) Transcript levels of *SmbHLH65* in the EV, ATCC, SmWRKY32O, and SmWRKY32R lines. (B and C) Y1H and EMSA experiments validated the binding between SmWRKY32 and the promoter of *SmbHLH65*. m, mutant. (D) Dual-LUC assays showed that SmWRKY32 represses the transactivation activity of the *SmbHLH65* promoter. Error bars represent SD (Student’s *t*-test, ^**^P < 0.01).

Previous research identified SmWRKY32 as a negative regulator of tanshinone synthesis in *S. miltiorrhiza* [23]*.* In the *SmWRKY32* overexpressing line transcriptome (accession number PRJNA1111010), the transcription factor *SmbHLH65* was significantly downregulated. To further investigate, qRT-PCR analysis was employed to assess the transcript level of *SmbHLH65* in *SmWRKY32* transgenic lines. This analysis revealed that the *SmbHLH65* transcript was markedly suppressed in the overexpression lines, while there was no notable change in the RNAi lines (Fig. 7A). Y1H experiments were employed to verify whether SmWRKY32 can bind to the *SmbHLH65* promoter. The Y1H results indicated that SmWRKY32 directly binds to the W-box (TTGACC) element of the *SmbHLH65* (−1474 to −1461 bp upstream of ATG) promoter (Fig. 7B). EMSA experiments were conducted to further investigate this result. In the EMSA assays, no shifted bands were observed when the GST protein was mixed with biotin-labeled probes containing the W-box element. However, shifted bands appeared in the existence of GST-SmWRKY32 and these probes. The introduction of a 50-fold excess of unlabeled cold probes eliminated the shifted bands, whereas mutant unlabeled cold probes at the same concentration had no effect (Fig. 7C). Dual-LUC assays were performed to evaluate the relative LUC activity of the *SmbHLH65* promoter regulated by SmWRKY32. The results indicated that SmWRKY32 substantially downregulated *SmbHLH65* expression by 2.4 times (Fig. 7D). These results confirm that SmWRKY32 directly represses *SmbHLH65* expression by binding to its promoter.

## Discussion

Tanshinones, valuable bioactive compounds in *S. miltiorrhiza*, are renowned for their cardiovascular protective effects. Their low content and unstable supply have prompted extensive research on their transcriptional regulatory networks. Transcription factors are pivotal in regulating tanshinone biosynthesis. For instance, SmSCR1, SmMYC2b, and SmERF73 have been identified as positive regulatory factors [45–47], while SmbZIP1 and SmbHLH3 serve as negative regulatory factors [48, 49].

This study identified the transcription factor SmbHLH65 from previous transcriptome sequencing analyses of *SmWRKY32*. Tissue-specific expression analysis in *S. miltiorrhiza* revealed that *SmbHLH65* is highly expressed in the stem and root (Fig. 1B). Xu *et al.* [50] demonstrated that tanshinones are synthesized and accumulated in the root, suggesting that SmbHLH65 may play a role in their biosynthesis. Transgenic analysis further indicated that SmbHLH65 is a positive regulator of tanshinone synthesis in *S. miltiorrhiza* (Fig. 2A). In *SmbHLH65* overexpression lines, the expression of key tanshinone biosynthesis genes was upregulated (Fig. 2B), potentially explaining the increased tanshinone accumulation. However, Y1H assays revealed that SmbHLH65 does not directly bind to the promoters of genes involved in the tanshinone biosynthesis pathway (Fig. S2). This phenomenon has also been observed in other plants. For instance, AaMYB108 does not directly bind to key enzyme genes associated with artemisinin biosynthesis but regulates synthesis by interacting with the positive regulator AaGSW1 in *Artemisia annua* [51]. Similarly, IbNAC29 does not bind to the promoters of carotenoid biosynthesis genes but forms a complex with IbMYB1R1 and IbAITR5 to promote carotenoid accumulation in sweet potatoes [52]. We speculate that SmbHLH65 may regulate tanshinone synthesis by interacting with other transcription factors.

Through screening the *S. miltiorrhiza* yeast two-hybrid library, we identified the SmbHLH85 protein. The interaction between SmbHLH65 and SmbHLH85 was subsequently validated through Y2H, LCI, and pull-down experiments (Fig. 3). Genetic analysis of SmbHLH85 revealed its positive role in tanshinone biosynthesis (Fig. 4A). Overexpressing *SmbHLH85* markedly increased *SmDXS2*, *SmDXR*, *SmGGPPS1*, *SmCPS1*, *SmKSL1*, *SmCYP76AH1*, *SmCYP76AH3*, *SmCYP76AK1*, and *SmCYP76AK2* expression. Conversely, silencing *SmbHLH85* resulted in a pronounced downregulation of these genes, as well as *SmCYP76AK3* (Fig. 4B). These results suggest that the significantly regulated enzyme genes may be direct targets of SmbHLH85. Y1H assays further confirmed that SmbHLH85 binds exclusively to the promoters of *SmDXS2* and *SmCPS1* (Fig. 5A and Fig. S4). A previous study reported that SmbHLH10 increases tanshinone yield by activating *SmDXS2*, *SmCPS1*, and *SmCPS5* expression [53]. Additionally, SmMYB36 enhances tanshinone biosynthesis by upregulating *SmDXS2*, *SmCPS1*, and *SmGGPPS1* [54]. EMSA and Dual-LUC assays confirmed that SmbHLH85 binds to the *SmDXS2* and *SmCPS1* promoters, activating their transcription (Fig. 5B and C). These findings suggest that SmbHLH85 improves tanshinone content by promoting the transcript of *SmDXS2* and *SmCPS1*.

Transcriptional complexes play a crucial role in coordinating the regulation of secondary metabolism biosynthesis in plants. For example, the interaction between AtBZR1 and AtPAP1 synergistically regulates the expression of AtPAP1 target genes, increasing anthocyanin biosynthesis in *Arabidopsis* [55]. In apples, the MdNAC1–MdbZIP23 complex enhances the transcription of *MdMYB10* and *MdUFGT*, promoting anthocyanin accumulation [56]. Similarly, MabHLH11 interacts with MaMYB4, strengthening MabHLH11's transcriptional activation of *MaUGT79* and enhancing scopolin accumulation in *Melilotus albus* [57]. SmMYB36 interacts with SmMYC2 to enhance the transactivation activity of SmMYC2 on *SmGGPPS1*, thereby stimulating tanshinone synthesis in *S. miltiorrhiza* [6]. Given the interaction between SmbHLH65 and SmbHLH85, we speculated that this interaction could influence SmbHLH85's binding and regulation of its target genes. Our results proved that the interaction between SmbHLH65 and SmbHLH85 heightened the binding of SmbHLH85 to the *SmDXS2* and *SmCPS1* promoters, further strengthening its activation of these genes (Fig. 6A and B). These findings indicate that SmbHLH65 regulates the accumulation of tanshinones by forming a complex with SmbHLH85. Although previous studies have investigated the function of transcription complexes in regulating secondary metabolism, research on their involvement in tanshinone biosynthesis is still limited, particularly regarding the regulation by bHLH heterodimers. Our research reveals that the SmbHLH65–SmbHLH85 complex synergistically regulates tanshinone biosynthesis by enhancing the transactivation activity of the target genes *SmDXS2* and *SmCPS1*. This finding offers promising avenues for further exploration of the broader functions of bHLH–bHLH complexes. Additionally, Nie *et al.* [23] previously reported that SmWRKY32 functions as a negative regulator in tanshinone synthesis. Here, we confirmed that SmWRKY32 binds to the *SmbHLH65* promoter, inhibiting its transcription. This suggests that SmWRKY32 may indirectly suppress tanshinone synthesis by downregulating *SmbHLH65* expression*.*

**Figure 8:**
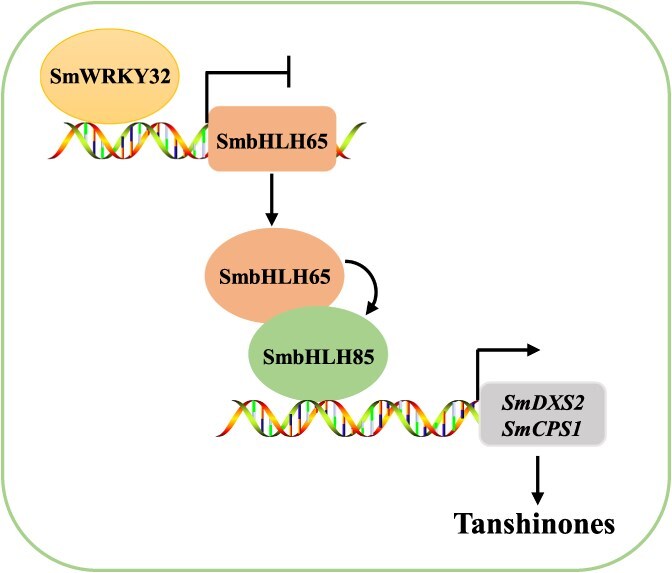
A working model of the SmWRKY32-SmbHLH65/SmbHLH85 regulation of tanshinone biosynthesis. SmWRKY32 binds to the *SmbHLH65* promoter, suppressing its expression. SmbHLH65 positively regulates tanshinone biosynthesis by forming a complex with SmbHLH85, which promotes synthesis by increasing *SmDXS2* and *SmCPS1* transcription. Additionally, SmbHLH65 stimulates the expression of *SmbHLH85*. SmWRKY32 indirectly inhibits tanshinone biosynthesis by suppressing the activation complex formed by SmbHLH65 and SmbHLH85.

In summary, we propose that the regulatory module SmWRKY32-SmbHLH65/SmbHLH85 participates in tanshinone biosynthesis in *S. miltiorrhiza* (Fig. 8). Both SmbHLH65 and SmbHLH85 serve as positive regulators of this biosynthetic pathway. Specifically, SmbHLH85 promotes the accumulation of tanshinones by directly activating *SmDXS2* and *SmCPS1*, while SmbHLH65 modulates this process through its interaction with SmbHLH85 and positively regulates the expression of *SmbHLH85*. Furthermore, *SmbHLH65* is regulated by the transcription factor SmWRKY32, which inhibits tanshinone biosynthesis.

## Materials and methods

### Plant materials, gene cloning, and bioinformatics analysis

The wild *S. miltiorrhiza* stored in our laboratory was used for tissue-specific expression analysis. Seedlings of *S. miltiorrhiza* and *N. benthamiana* were grown in a greenhouse. The hairy roots were cultured as previously reported [23, 58]. The ORFs of *SmbHLH65* and *SmbHLH85* were cloned from the cDNA of *S. miltiorrhiza* using specific primers (Table S1). DNAMAN version 6.0 software was used for protein sequence alignment analysis.

### RNA extraction and qRT-PCR

Total RNA was extracted using the RNA Extraction Kit (AG21022; Accurate Biology, Hunan, China). Following reverse transcription to cDNA with the Reverse Transcription Kit (AG11728, Accurate Biology), qRT-PCR analysis was conducted with the qPCR Kit (AG11701, Accurate Biology). Relative gene expression was calculated using the 2^–△△CT^ method. *SmActin* was used as the internal control. All analyses were three replicates. The primers used for this study are shown in Table S1.

### Subcellular localization

The coding sequences (without termination codons) of *SmbHLH65* and *SmbHLH85* were independently cloned into the empty pCsGFPBT plasmid, which carries the *GFP* reporter gene. The fusion constructs pCsGFPBT-SmbHLH65, pCsGFPBT-SmbHLH85, or pCsGFPBT, along with pCambia1300-mCherry-SmWRKY2, were transiently transferred into tobacco through *Agrobacterium tumefaciens* GV3101 containing the helper plasmid pSoup19. Two days later, the fluorescence was checked with an Inverted Fluorescence Microscope (DMi8, Leica, Germany). The pCsGFPBT empty vector acted as the negative control, and nuclei were labeled using pCambia1300-mCherry-SmWRKY2. The excitation wavelengths were 470 nm for GFP and 530 nm for mCherry.

### Creation of transgenic and determination of tanshinone content

The transgenic hairy roots were obtained from the infection of *S. miltiorrhiza* leaves with *Agrobacterium rhizogenes* ATCC15834 harboring the plasmids pK7WG2R-SmbHLH65 (SmbHLH65O), pK7WG2R-SmbHLH85 (SmbHLH85O), pK7GWIWG2R-SmbHLH65 (SmbHLH65R), pK7GWIWG2R-SmbHLH85 (SmbHLH85R), and pK7WG2R-EV, following the transformation process described by Pei *et al.* [59]. Genomic DNA PCR amplification was carried out to screen for positive hairy roots. The methods for tanshinone extraction and detection followed previous research [6, 23].

### Yeast two-hybrid assay

The ORF of *SmbHLH65* was cloned into the pGBKT7 vector. pGBKT7-SmbHLH65 was used as bait to screen potential interacting proteins in a Danshen cDNA library. *SmNAC2*, *SmAGL14*, *SmbHLH37*, and *SmbHLH85* cDNA sequences were individually integrated into the pGADT7 vector. The experimental group (pGADT7-SmNAC2/SmAGL14/SmbHLH37/SmbHLH85 and pGBKT7-SmbHLH65) and the negative controls (pGBKT7 and pGADT7; pGBKT7-SmbHLH65 and pGADT7; pGBKT7 and pGADT7-SmNAC2/SmAGL14/SmbHLH37/SmbHLH85) were transferred into yeast Y2H following previous research [58, 60].

### LCI assay

LCI assays were performed following the previous method [60, 61]. The cDNAs of *SmbHLH65* (without the stop codon) and *SmbHLH85* were inserted into the pCAMBIA-nLUC (JW771) and pCAMBIA-cLUC (JW772) vectors, respectively. The recombinants SmbHLH65-nLUC, cLUC-SmbHLH85, and two types of empty vector plasmids were transformed into *A. tumefaciens* GV3101. The combinations of SmbHLH65-nLUC and cLUC-SmbHLH85, SmbHLH65-nLUC and cLUC, nLUC and cLUC-SmbHLH85, and nLUC and cLUC were injected into different regions of the same tobacco leaf. After overnight incubation in the dark, the samples were exposed to light for 2 days. Beetle luciferin potassium salt (E1601; Promega [Beijing] Biotech, Beijing, China) was applied to the injection sites, and the samples were kept in the dark for 5 min. LUC activity was measured using a CCD imaging camera (Lumazone Pylon 2048B, Princeton, NJ).

### Pull-down assay

*SmbHLH65* and *SmbHLH85* ORFs without the stop codon were separately ligated into the pET32a and pGEX-4 T-1 plasmids. The GST protein, GST-SmbHLH85, and His-SmbHLH65 fusion proteins were induced and purified on the basis of previous research [58]. Pull-down assays were performed using GST-tag Purification Resin (P2250; Beyotime, Shanghai, China) following the manufacturer's instructions with modifications. In the assay, purified His-SmbHLH65 and either GST or GST-SmbHLH85 proteins were incubated at 4°C for 1 h, and a portion of the supernatant was retained as input. The remaining mixture was then incubated with GST beads at 4°C for 2 h. After incubation, the beads were washed, and the pulled-down proteins were detected by Western blotting using GST antibody (Beyotime) and His antibody (TransGen, Beijing, China).

### Yeast one-hybrid assay

*SmWRKY32*, *SmbHLH65*, and *SmbHLH85* ORFs were separately cloned into the pB42AD plasmid. Additionally, three copies of the W-box in the *SmbHLH65* promoter and three copies of the E/G-box in *SmDXS2*, *SmDXR*, *SmGGPPS1*, *SmCPS1*, *SmKSL1*, *SmCYP76AH1*, *SmCYP76AH3*, *SmCYP76AK1*, *SmCYP76AK2*, and *SmCYP76AK3* were cloned into the pLacZi plasmid. The pB42AD and pLacZi recombinants for each combination were cotransformed into EGY48 yeast strains following previous research [6] and cultured on SD/−Ura/−Trp medium for 3 days. Positive clones were observed to turn blue on SD/−Ura/−Trp medium supplemented with X-gal.

### Electrophoretic mobility shift assay

The probes used in this analysis were synthesized at Qingke Biotech Company (Beijing, China) and prepared by annealing complementary oligonucleotides at 95°C for 5 min and then at 70°C for 20 min. EMSAs were conducted following the chemiluminescent EMSA Kit (GS009, Beyotime) described approach. The purified protein and either unlabeled or mutated cold probes were incubated in EMSA/Gel-Shift binding buffer at 25°C for 10 min, followed by incubation with biotin-labeled probes for 20 min. The resulting mixture was separated by 6% polyacrylamide gel electrophoresis and detected using a chemiluminescence gel imaging system (ChemiDoc XRS+; Bio-Rad, Hercules, CA). The probes employed for the EMSA experiment are detailed in Table S1.

### Dual-luciferase assay

*SmDXS2*, *SmCPS1*, and *SmbHLH65* promoters were separately ligated into the pGreenII 0800-LUC plasmid to create the reporter recombinants. The effectors included pCsGFPBT-SmbHLH65, pCsGFPBT-SmbHLH85, and pCsGFPBT-WRKY32, while the pCsGFPBT plasmid served as the effector control. Both the effectors and reporters were separately transformed into GV3101 and cotransfected into tobacco. After a 20-h dark culture followed by 48 h of light culture, LUC and REN activities were measured with the TransDetect Double-Luciferase Reporter Assay Kit (FR201, TransGen). The dual-luciferase assay was carried out as described by Liu *et al.* [62]. The LUC/REN ratio was expressed as the relative luciferase activity.

### Statistical analysis

Student's *t*-test or ordinary one-way ANOVA with multiple comparisons was used to determine statistical significance. Significant differences are indicated by ^**^P < 0.01 and ^*^P < 0.05.

## Supplementary Material

Web_Material_uhaf096

## Data Availability

The data that support the results of this study can be found in this paper and its supplementary materials.
