# Supplementary material for: The SmWRKY32-SmbHLH65/SmbHLH85 regulatory module mediates tanshinone biosynthesis in Salvia miltiorrhiza
Source: Hortic Res. 2025 Mar 25;12(7):uhaf096. doi: 10.1093/hr/uhaf096 (PMC12087451; doi:10.1093/hr/uhaf096)
Supplement: Web_Material_uhaf096 [file web_material_uhaf096.docx]

**
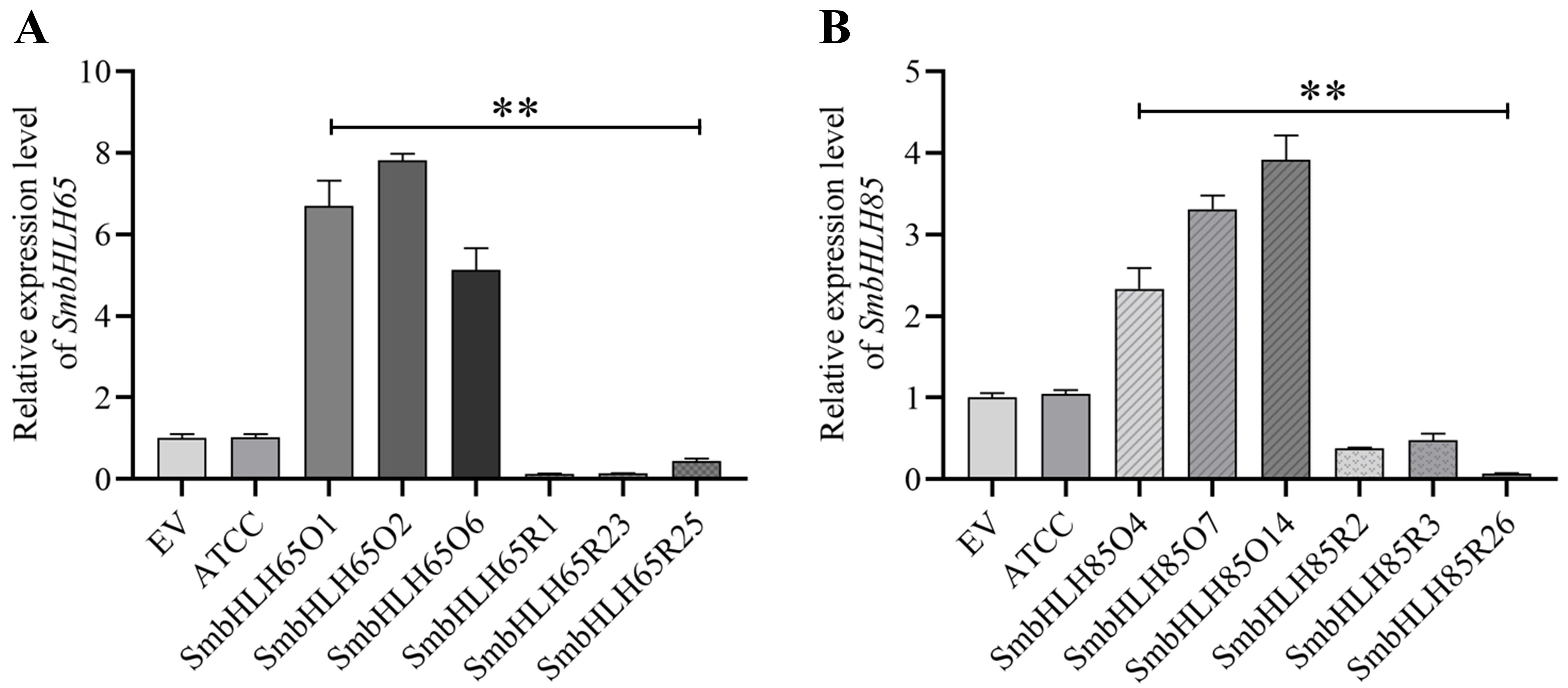
**

Figure S1. Relative expression levels of *SmbHLH65* and *SmbHLH85* in their respective transgenic hairy root lines. Values are presented as means ± SD of three replicates. (Student’s t-test, ***P* < 0.01).

**
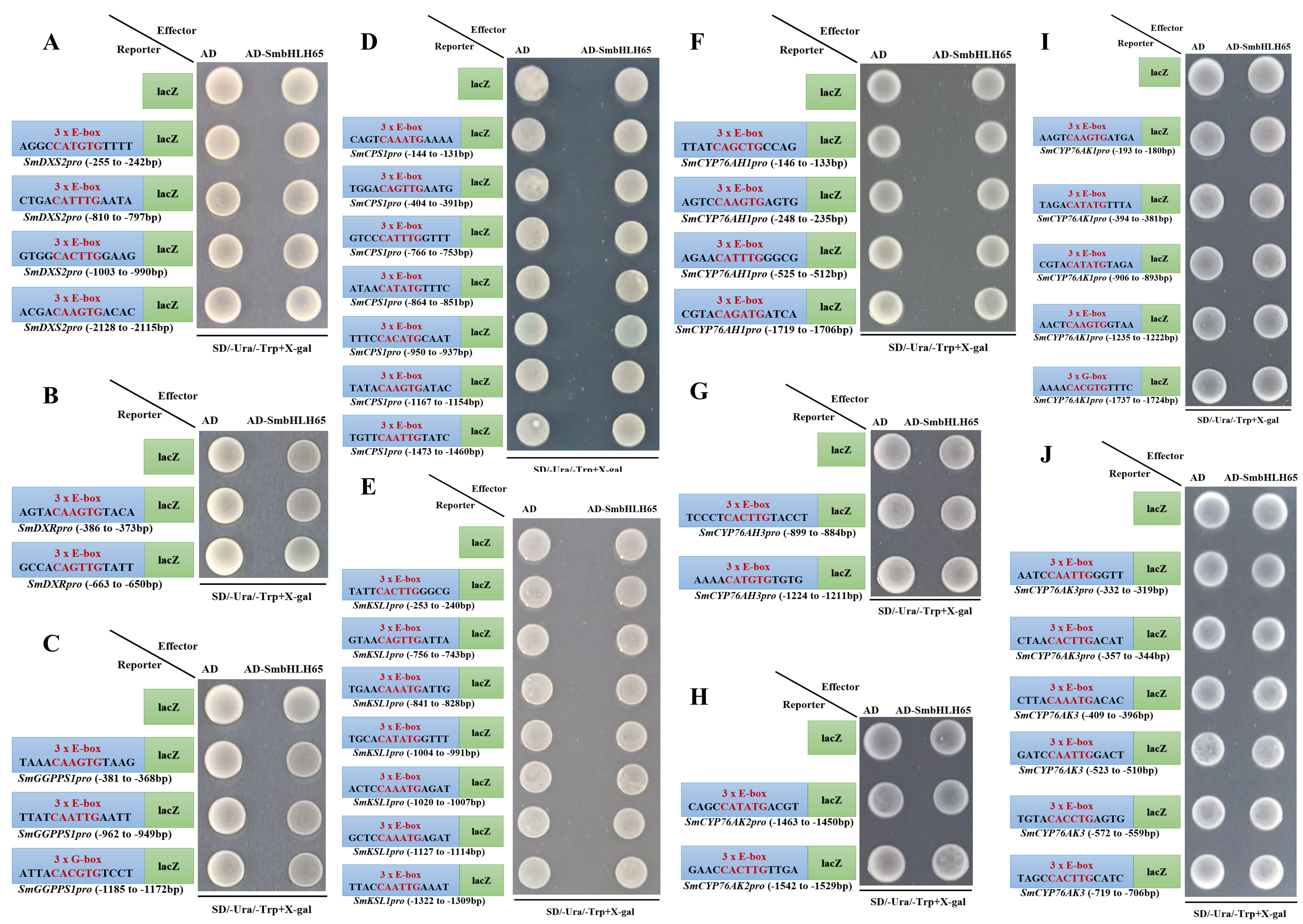
**

Figure S2. Y1H assays demonstrating that SmbHLH65 cannot bind to the promoters of key enzyme genes in the tanshinone biosynthesis pathway.

**
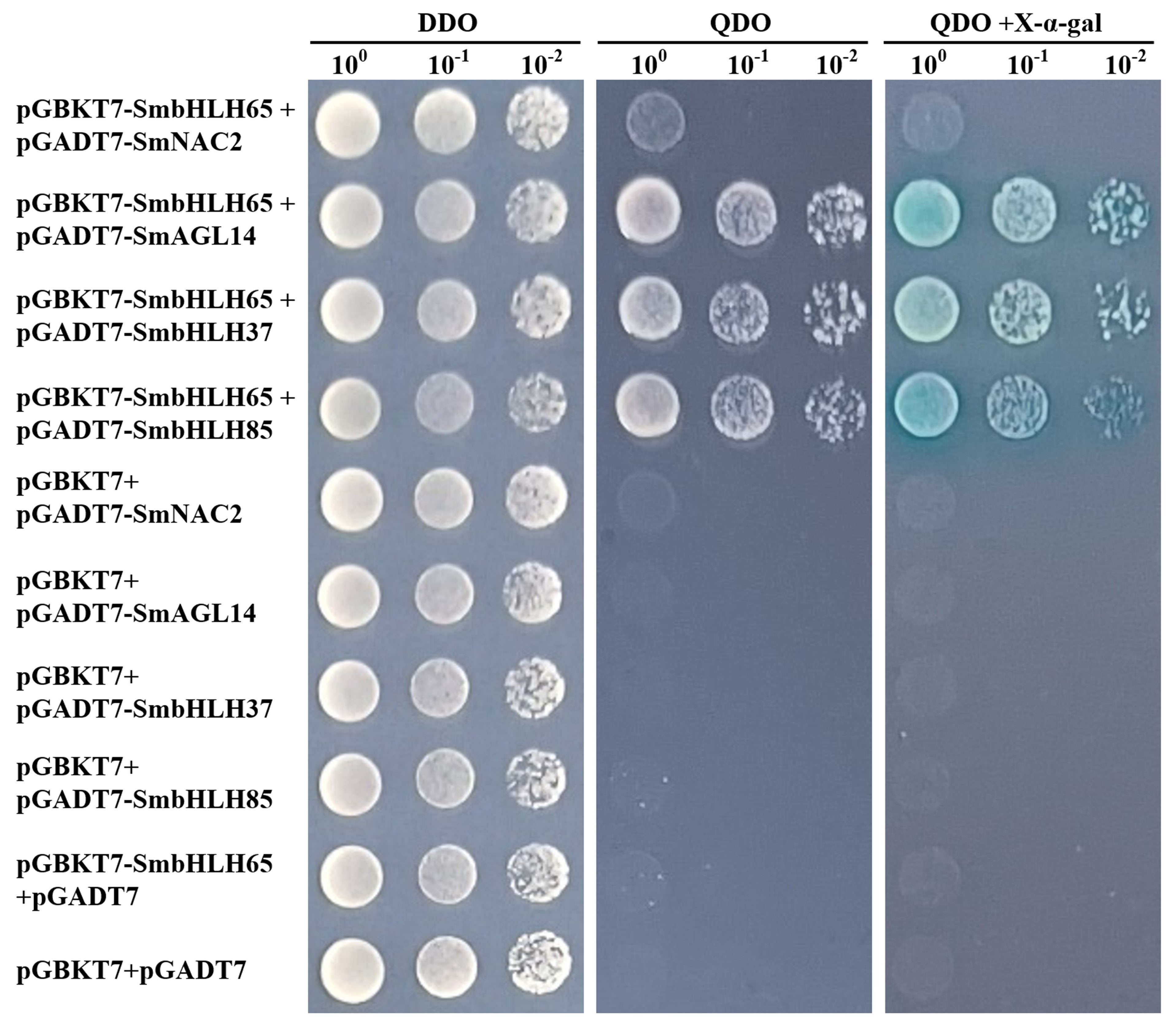
**

Figure S3. Yeast two-hybrid screening for proteins that interact with SmbHLH65.


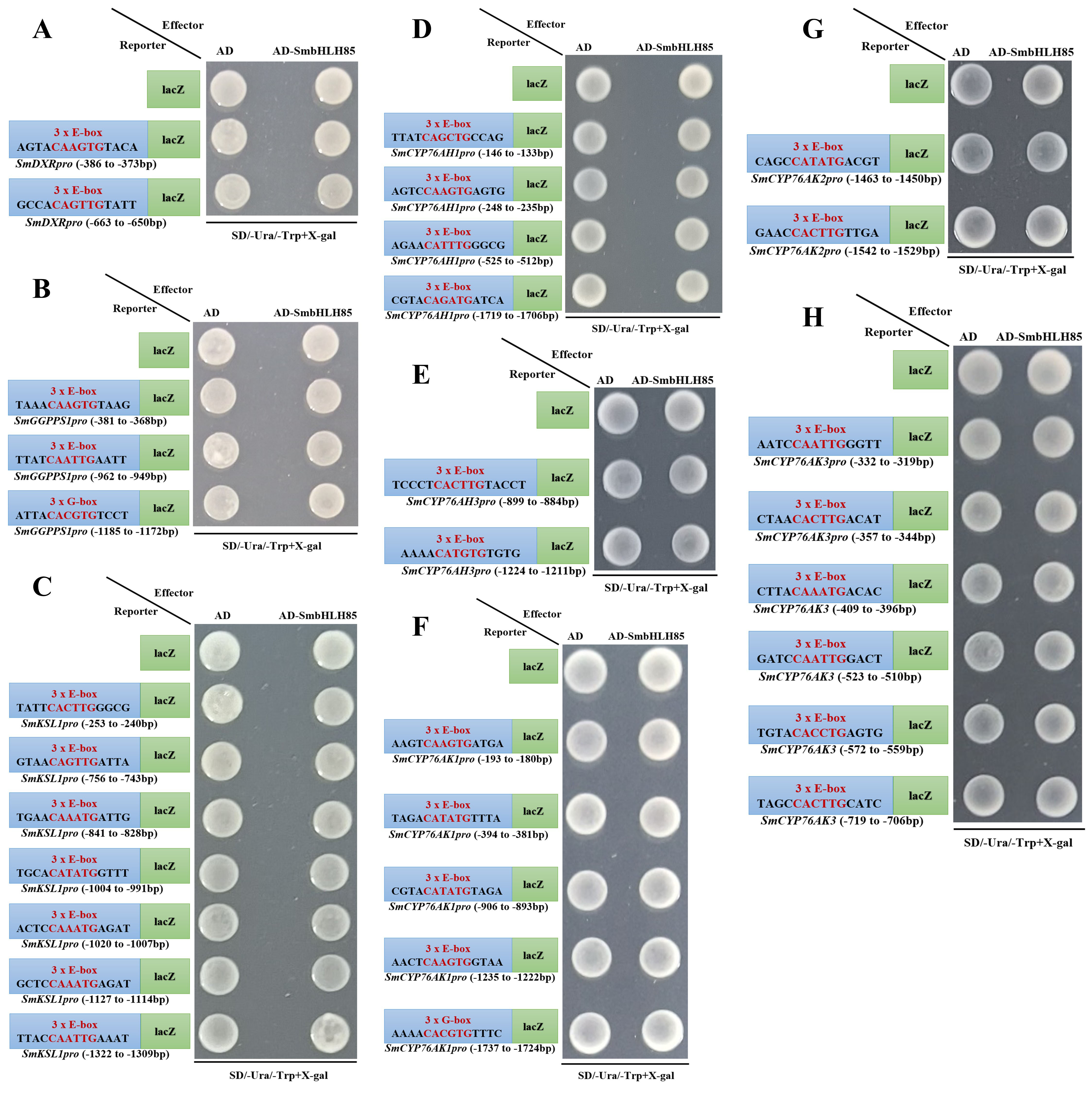


Figure S4. Y1H assays confirming that SmbHLH85 cannot bind to the promoters of these tanshinone biosynthesis genes.

**
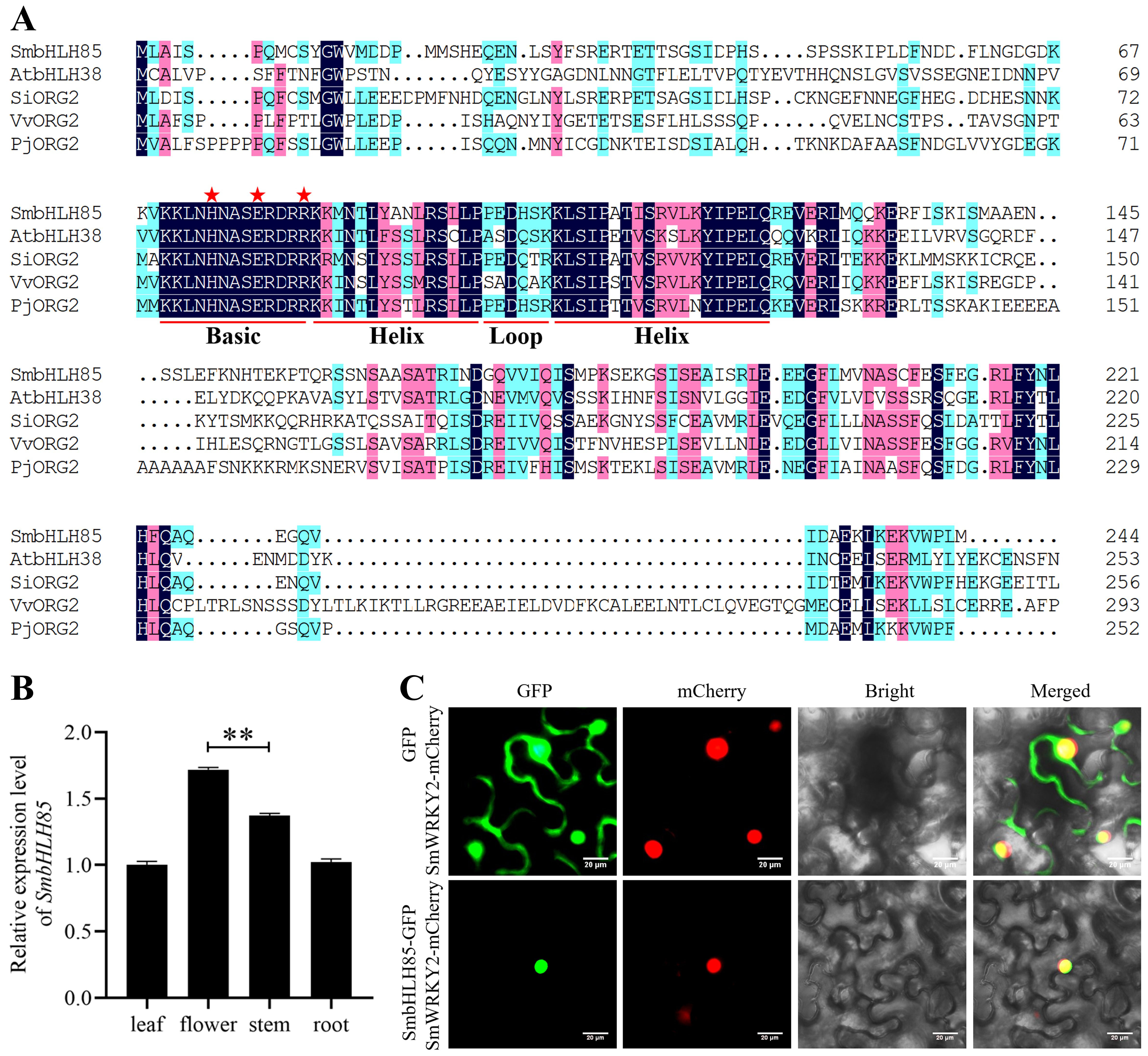
**

Figure S5. Molecular characterization and subcellular localization of SmbHLH85. (A) Multiple sequence alignment of SmbHLH85 with bHLH proteins from various plant species, including AtbHLH38 (AT3G56970.1), SiORG2 (XP_011075620.1), VvORG2 (RVW34500.1), and PjORG2 (GFP80820.1). The bHLH domain and HER motif are highlighted by red lines and red pentagrams, respectively. (B) Relative expression levels of *SmbHLH85* in different tissues of two-year-old *S. miltiorrhiza.* The leaf tissue was used as the control. Error bars indicate SD (Student’s t-test, ***P* < 0.01). (C) Subcellular localization of SmbHLH85.

**Table S1. All primers used in this study**

| **Primer Name** | **Primer sequence (5'-3')** |
| --- | --- |
|  | **Gene Clone** |
| SmbHLH65F | ATGCAGCAAGAAAACAGTGGC |
| SmbHLH65R | TCAAAACGACCTGTAGTGATCAAG |
| SmbHLH85F | ATGTTGGCTATTTCTCCTCAAATG |
| SmbHLH85R | CTACATCAAAGGCCAAACTTTCTC |
| SmNAC2F | ATGGTAGGATCAAGAATCAGCTCT |
| SmNAC2R | CTACTGAAACTGATCATAAACCGGA |
| SmAGL14F | ATGGTGAGGGGAAAAACTGAG |
| SmAGL14R | TTAGGTAATTGAAAAACTGTAAATTGAG |
| SmbHLH37F | ATGGGAAAGAAAGTATGGTG |
| SmbHLH37R | TCATTTCAAGAGAGCAGCAG |
|  | **Transgenic plant** |
| pK7WG2R-SmbHLH65F | GGGGACAAGTTTGTACAAAAAAGCAGGCTTAATGCAGCAAGAAAACAGTGGC |
| pK7WG2R-SmbHLH65R | GGGGACCACTTTGTACAAGAAAGCTGGGTATCAAAACGACCTGTAGTGATCAAG |
| pK7GWIWG2R-SmbHLH65F | GGGGACAAGTTTGTACAAAAAAGCAGGCTTAAATCACTACCTATTATAGAAACCTCGG |
| pK7GWIWG2R-SmbHLH65R | GGGGACCACTTTGTACAAGAAAGCTGGGTAGATACATTAATTTGGTGATCCTCACAT |
| pK7WG2R-SmbHLH85F | GGGGACAAGTTTGTACAAAAAAGCAGGCTTAATGTTGGCTATTTCTCCTCAAATG |
| pK7WG2R-SmbHLH85R | GGGGACCACTTTGTACAAGAAAGCTGGGTACTACATCAAAGGCCAAACTTTCTC |
| pK7GWIWG2R-SmbHLH85F | GGGGACAAGTTTGTACAAAAAAGCAGGCTTAAAGTGATAGATGCTGAGAAGTTGAAG |
| pK7GWIWG2R-SmbHLH85R | GGGGACCACTTTGTACAAGAAAGCTGGGTAACCTCTTGTTCTATTCCATTAATGTCT |
| 35S-F | GACGCACAATCCCACTATCC |
| rolB-F | GCTCTTGCAGTGCTAGATTT |
| rolB-R | GAAGGTGCAAGCTACCTCTC |
| rolC-F | CTCCTGACATCAAACTCGTC |
| rolC-R | TGCTTCGAGTTATGGGTACA |
|  | **qRT-PCR** |
| SmActin-qF | GGTGCCCTGAGGTCCTGTT |
| SmActin-qR | AGGAACCACCGATCCAGACA |
| SmbHLH65-qF | AGCAAGAAAACAGTGGCGATG |
| SmbHLH65-qR | GAGAAACGGGTCGTGGTCG |
| SmbHLH85-qF | ACACTCTGTATGCCAACCTTCG |
| SmbHLH85-qR | AGATGGTGGCTGGAATGCTC |
| SmDXS2-qF | CTCACGGTCGCATTGCATCAT |
| SmDXS2-qR | CGCTTTCGTCTCGTTTAGGGA |
| SmDXR-qF | CATGCGTTTGCCTATTCTGTAC |
| SmDXR-qR | ACTAAGAACTCCGGTCATGGTG |
| SmGGPPS1-qF | ACAAGACCACGTATCCCAAGC |
| SmGGPPS1-qR | TCTGCCTATGTGCAATGTAATCG |
| SmCPS1-qF | CCACATCGCCTTCAGGGAAGAAAT |
| SmCPS1-qR | TTTATGCTCGATTTCGCTGCGATCT |
| SmKSL1-qF | TGGAAACAGTGTGACCCTTCTGCT |
| **Primer Name** | **Primer sequence (5'-3')** |
| SmKSL1-qR | GCTTGCATACAAATAACACCCAATCCT |
| SmCYP76AH1-qF | ACGCATCACTTCACCCATCTCA |
| SmCYP76AH1-qR | ATTGCCGACTCATCCACGAT |
| SmCYP76AH3-qF | CCCCAACTTCGCCGACTACTTC |
| SmCYP76AH3-qR | CAGCCTGGGCATGAGCGACT |
| SmCYP76AK1-qF | CCACCAACATGGGCGTTCC |
| SmCYP76AK1-qR | TGTCGGATTCCTCCACGATGCT |
| SmCYP76AK2-qF | GCTTATCACTTCGGGAGTTTGC |
| SmCYP76AK2-qR | TCGTCAATGCTCAAATCATACTCA |
| SmCYP76AK3-qF | CGGCTGCGAGCAAGAAAGGA |
| SmCYP76AK3-qR | GTTGTGGCTGCGCTTGTGTC |
|  | **LCI** |
| nLUC-SmbHLH65F | CACGGGGGACGAGCTCGGTACCATGCAGCAAGAAAACAGTGGC |
| nLUC-SmbHLH65R | ACGCGTACGAGATCTGGTCGACAAACGACCTGTAGTGATCAAG |
| cLUC-SmbHLH85F | ACGCGTCCCGGGGCGGTACCATGTTGGCTATTTCTCCTCAAATG |
| cLUC-SmbHLH85R | acgaaagctctgcaggtcgacCTACATCAAAGGCCAAACTTTCTC |
|  | **Y2H** |
| pGBKT7-SmbHLH65F | ATGGCCATGGAGGCCGAATTCATGCAGCAAGAAAACAGTGGC |
| pGBKT7-SmbHLH65R | CCGCTGCAGGTCGACGGATCCTCAAAACGACCTGTAGTGATCAAG |
| pGADT7-SmbHLH85F | ATGGAGGCCAGTGAATTCATGTTGGCTATTTCTCCTCAAATG |
| pGADT7-SmbHLH85R | CCCACCCGGGTGGAATTCCTACATCAAAGGCCAAACTTTCTC |
| pGADT7-SmNAC2F | ATGGAGGCCAGTGAATTCATGGTAGGATCAAGAATCAGCT |
| pGADT7-SmNAC2R | CCCACCCGGGTGGAATTCCTACTGAAACTGATCATAAACC |
| pGADT7-SmAGL14F | ATGGAGGCCAGTGAATTCATGGTGAGGGGAAAAACT |
| pGADT7-SmAGL14R | CCCACCCGGGTGGAATTCTTAGGTAATTGAAAAACTGTAAAT |
| pGADT7-SmbHLH37F | ATGGAGGCCAGTGAATTCATGGGAAAGAAAGTATGGTG |
| pGADT7-SmbHLH37R | CCCACCCGGGTGGAATTCTCATTTCAAGAGAGCAGCAG |
|  | **Y1H** |
| pB42AD-SmbHLH65F | gattatgcctctcccgaattcATGCAGCAAGAAAACAGTGGC |
| pB42AD-SmbHLH65R | agaagtccaaagcttctcgagTCAAAACGACCTGTAGTGATCAAG |
| pB42AD-SmbHLH85F | gattatgcctctcccgaattcATGTTGGCTATTTCTCCTCAAATG |
| pB42AD-SmbHLH85R | agaagtccaaagcttctcgagCTACATCAAAGGCCAAACTTTCTC |
| pB42AD-SmWRKY32F | gattatgcctctcccgaattcATGGAGGATGATCGAAGAATAGTAGA |
| pB42AD-SmWRKY32R | agaagtccaaagcttctcgagTTAGAAGGAAGAGTAAATTTGCATCTG |
| pLacZi-pSmDXS2-F1 | AATTCGAGGCCATGTGTTTTAGGCCATGTGTTTTAGGCCATGTGTTTTACC |
| pLacZi-pSmDXS2-R1 | TCGAGGTAAAACACATGGCCTAAAACACATGGCCTAAAACACATGGCCTCG |
| pLacZi-pSmDXS2-F2 | AATTCGCTGACATTTGAATACTGACATTTGAATACTGACATTTGAATAACC |
| pLacZi-pSmDXS2-R2 | TCGAGGTTATTCAAATGTCAGTATTCAAATGTCAGTATTCAAATGTCAGCG |
| pLacZi-pSmDXS2-F3 | AATTCGGTGGCACTTGGAAGGTGGCACTTGGAAGGTGGCACTTGGAAGACC |
| pLacZi-pSmDXS2-R3 | TCGAGGTCTTCCAAGTGCCACCTTCCAAGTGCCACCTTCCAAGTGCCACCG |
| pLacZi-pSmDXS2-F4 | AATTCGACGACAAGTGACACACGACAAGTGACACACGACAAGTGACACACC |
| pLacZi-pSmDXS2-R4 | TCGAGGTGTGTCACTTGTCGTGTGTCACTTGTCGTGTGTCACTTGTCGTCG |
| pLacZi-pSmDXR-F1 | AATTCGAGTACAAGTGTACAAGTACAAGTGTACAAGTACAAGTGTACAACC |
| **Primer Name** | **Primer sequence (5'-3')** |
| pLacZi-pSmDXR-R1 | TCGAGGTTGTACACTTGTACTTGTACACTTGTACTTGTACACTTGTACTCG |
| pLacZi-pSmDXR-F2 | AATTCGGCCACAGTTGTATTGCCACAGTTGTATTGCCACAGTTGTATTACC |
| pLacZi-pSmDXR-R2 | TCGAGGTAATACAACTGTGGCAATACAACTGTGGCAATACAACTGTGGCCG |
| pLacZi-pSmGGPPS1-F1 | AATTCGTAAACAAGTGTAAGTAAACAAGTGTAAGTAAACAAGTGTAAGACC |
| pLacZi-pSmGGPPS1-R1 | TCGAGGTCTTACACTTGTTTACTTACACTTGTTTACTTACACTTGTTTACG |
| pLacZi-pSmGGPPS1-F2 | AATTCGTTATCAATTGAATTTTATCAATTGAATTTTATCAATTGAATTACC |
| pLacZi-pSmGGPPS1-R2 | TCGAGGTAATTCAATTGATAAAATTCAATTGATAAAATTCAATTGATAACG |
| pLacZi-pSmGGPPS1-F3 | AATTCGATTACACGTGTCCTATTACACGTGTCCTATTACACGTGTCCTACC |
| pLacZi-pSmGGPPS1-R3 | TCGAGGTAGGACACGTGTAATAGGACACGTGTAATAGGACACGTGTAATCG |
| pLacZi-pSmCPS1-F1 | AATTCGCAGTCAAATGAAAACAGTCAAATGAAAACAGTCAAATGAAAAACC |
| pLacZi-pSmCPS1-R1 | TCGAGGTTTTTCATTTGACTGTTTTCATTTGACTGTTTTCATTTGACTGCG |
| pLacZi-pSmCPS1-F2 | AATTCGTGGACAGTTGAATGTGGACAGTTGAATGTGGACAGTTGAATGACC |
| pLacZi-pSmCPS1-R2 | TCGAGGTCATTCAACTGTCCACATTCAACTGTCCACATTCAACTGTCCACG |
| pLacZi-pSmCPS1-F3 | AATTCGGTCCCATTTGGTTTGTCCCATTTGGTTTGTCCCATTTGGTTTACC |
| pLacZi-pSmCPS1-R3 | TCGAGGTAAACCAAATGGGACAAACCAAATGGGACAAACCAAATGGGACCG |
| pLacZi-pSmCPS1-F4 | AATTCGATAACATATGTTTCATAACATATGTTTCATAACATATGTTTCACC |
| pLacZi-pSmCPS1-R4 | TCGAGGTGAAACATATGTTATGAAACATATGTTATGAAACATATGTTATCG |
| pLacZi-pSmCPS1-F5 | AATTCGTTTCCACATGCAATTTTCCACATGCAATTTTCCACATGCAATACC |
| pLacZi-pSmCPS1-R5 | TCGAGGTATTGCATGTGGAAAATTGCATGTGGAAAATTGCATGTGGAAACG |
| pLacZi-pSmCPS1-F6 | AATTCGTATACAAGTGATACTATACAAGTGATACTATACAAGTGATACACC |
| pLacZi-pSmCPS1-R6 | TCGAGGTGTATCACTTGTATAGTATCACTTGTATAGTATCACTTGTATACG |
| pLacZi-pSmCPS1-F7 | AATTCGTGTTCAATTGTATCTGTTCAATTGTATCTGTTCAATTGTATCACC |
| pLacZi-pSmCPS1-R7 | TCGAGGTGATACAATTGAACAGATACAATTGAACAGATACAATTGAACACG |
| pLacZi-pSmKSL1-F1 | AATTCGTATTCACTTGGGCGTATTCACTTGGGCGTATTCACTTGGGCGACC |
| pLacZi-pSmKSL1-R1 | TCGAGGTCGCCCAAGTGAATACGCCCAAGTGAATACGCCCAAGTGAATACG |
| pLacZi-pSmKSL1-F2 | AATTCGGTAACAGTTGATTAGTAACAGTTGATTAGTAACAGTTGATTAACC |
| pLacZi-pSmKSL1-R2 | TCGAGGTTAATCAACTGTTACTAATCAACTGTTACTAATCAACTGTTACCG |
| pLacZi-pSmKSL1-F3 | AATTCGTGAACAAATGATTGTGAACAAATGATTGTGAACAAATGATTGACC |
| pLacZi-pSmKSL1-R3 | TCGAGGTCAATCATTTGTTCACAATCATTTGTTCACAATCATTTGTTCACG |
| pLacZi-pSmKSL1-F4 | AATTCGTGCACATATGGTTTTGCACATATGGTTTTGCACATATGGTTTACC |
| pLacZi-pSmKSL1-R4 | TCGAGGTAAACCATATGTGCAAAACCATATGTGCAAAACCATATGTGCACG |
| pLacZi-pSmKSL1-F5 | AATTCGACTCCAAATGAGATACTCCAAATGAGATACTCCAAATGAGATACC |
| pLacZi-pSmKSL1-R5 | TCGAGGTATCTCATTTGGAGTATCTCATTTGGAGTATCTCATTTGGAGTCG |
| pLacZi-pSmKSL1-F6 | AATTCGGCTCCAAATGAGATGCTCCAAATGAGATGCTCCAAATGAGATACC |
| pLacZi-pSmKSL1-R6 | TCGAGGTATCTCATTTGGAGCATCTCATTTGGAGCATCTCATTTGGAGCCG |
| pLacZi-pSmKSL1-F7 | AATTCGTTACCAATTGAAATTTACCAATTGAAATTTACCAATTGAAATACC |
| pLacZi-pSmKSL1-R7 | TCGAGGTATTTCAATTGGTAAATTTCAATTGGTAAATTTCAATTGGTAACG |
| pLacZi-pSmCYP76AH1-F1 | AATTCGTTATCAGCTGCCAGTTATCAGCTGCCAGTTATCAGCTGCCAGACC |
| pLacZi-pSmCYP76AH1-R1 | TCGAGGTCTGGCAGCTGATAACTGGCAGCTGATAACTGGCAGCTGATAACG |
| pLacZi-pSmCYP76AH1-F2 | AATTCGAGTCCAAGTGAGTGAGTCCAAGTGAGTGAGTCCAAGTGAGTGACC |
| pLacZi-pSmCYP76AH1-R2 | TCGAGGTCACTCACTTGGACTCACTCACTTGGACTCACTCACTTGGACTCG |
| pLacZi-pSmCYP76AH1-F3 | AATTCGAGAACATTTGGGCGAGAACATTTGGGCGAGAACATTTGGGCGACC |
| pLacZi-pSmCYP76AH1-R3 | TCGAGGTCGCCCAAATGTTCTCGCCCAAATGTTCTCGCCCAAATGTTCTCG |
| **Primer Name** | **Primer sequence (5'-3')** |
| pLacZi-pSmCYP76AH1-F4 | AATTCGCGTACAGATGATCACGTACAGATGATCACGTACAGATGATCAACC |
| pLacZi-pSmCYP76AH1-R4 | TCGAGGTTGATCATCTGTACGTGATCATCTGTACGTGATCATCTGTACGCG |
| pLacZi-pSmCYP76AH3-F1 | AATTCGTCCCTCACTTGTACCTTCCCTCACTTGTACCTTCCCTCACTTGTACCTACC |
| pLacZi-pSmCYP76AH3-R1 | TCGAGGTAGGTACAAGTGAGGGAAGGTACAAGTGAGGGAAGGTACAAGTGAGGGACG |
| pLacZi-pSmCYP76AH3-F2 | AATTCGAAAACATGTGTGTGAAAACATGTGTGTGAAAACATGTGTGTGACC |
| pLacZi-pSmCYP76AH3-R2 | TCGAGGTCACACACATGTTTTCACACACATGTTTTCACACACATGTTTTCG |
| pLacZi-pSmCYP76AK1-F1 | AATTCGAAGTCAAGTGATGAAAGTCAAGTGATGAAAGTCAAGTGATGAACC |
| pLacZi-pSmCYP76AK1-R1 | TCGAGGTTCATCACTTGACTTTCATCACTTGACTTTCATCACTTGACTTCG |
| pLacZi-pSmCYP76AK1-F2 | AATTCGTAGACATATGTTTATAGACATATGTTTATAGACATATGTTTAACC |
| pLacZi-pSmCYP76AK1-R2 | TCGAGGTTAAACATATGTCTATAAACATATGTCTATAAACATATGTCTACG |
| pLacZi-pSmCYP76AK1-F3 | AATTCGCGTACATATGTAGACGTACATATGTAGACGTACATATGTAGAACC |
| pLacZi-pSmCYP76AK1-R3 | TCGAGGTTCTACATATGTACGTCTACATATGTACGTCTACATATGTACGCG |
| pLacZi-pSmCYP76AK1-F4 | AATTCGAACTCAAGTGGTAAAACTCAAGTGGTAAAACTCAAGTGGTAAACC |
| pLacZi-pSmCYP76AK1-R4 | TCGAGGTTTACCACTTGAGTTTTACCACTTGAGTTTTACCACTTGAGTTCG |
| pLacZi-pSmCYP76AK1-F5 | AATTCGAAAACACGTGTTTCAAAACACGTGTTTCAAAACACGTGTTTCACC |
| pLacZi-pSmCYP76AK1-R5 | TCGAGGTGAAACACGTGTTTTGAAACACGTGTTTTGAAACACGTGTTTTCG |
| pLacZi-pSmCYP76AK2-F1 | AATTCGCAGCCATATGACGTCAGCCATATGACGTCAGCCATATGACGTACC |
| pLacZi-pSmCYP76AK2-R1 | TCGAGGTACGTCATATGGCTGACGTCATATGGCTGACGTCATATGGCTGCG |
| pLacZi-pSmCYP76AK2-F2 | AATTCGGAACCACTTGTTGAGAACCACTTGTTGAGAACCACTTGTTGAACC |
| pLacZi-pSmCYP76AK2-R2 | TCGAGGTTCAACAAGTGGTTCTCAACAAGTGGTTCTCAACAAGTGGTTCCG |
| pLacZi-pSmCYP76AK3-F1 | AATTCGAATCCAATTGGGTTAATCCAATTGGGTTAATCCAATTGGGTTACC |
| pLacZi-pSmCYP76AK3-R1 | TCGAGGTAACCCAATTGGATTAACCCAATTGGATTAACCCAATTGGATTCG |
| pLacZi-pSmCYP76AK3-F2 | AATTCGCTAACACTTGACATCTAACACTTGACATCTAACACTTGACATACC |
| pLacZi-pSmCYP76AK3-R2 | TCGAGGTATGTCAAGTGTTAGATGTCAAGTGTTAGATGTCAAGTGTTAGCG |
| pLacZi-pSmCYP76AK3-F3 | AATTCGCTTACAAATGACACCTTACAAATGACACCTTACAAATGACACACC |
| pLacZi-pSmCYP76AK3-R3 | TCGAGGTGTGTCATTTGTAAGGTGTCATTTGTAAGGTGTCATTTGTAAGCG |
| pLacZi-pSmCYP76AK3-F4 | AATTCGGATCCAATTGGACTGATCCAATTGGACTGATCCAATTGGACTACC |
| pLacZi-pSmCYP76AK3-R4 | TCGAGGTAGTCCAATTGGATCAGTCCAATTGGATCAGTCCAATTGGATCCG |
| pLacZi-pSmCYP76AK3-F5 | AATTCGTGTACACCTGAGTGTGTACACCTGAGTGTGTACACCTGAGTGACC |
| pLacZi-pSmCYP76AK3-R5 | TCGAGGTCACTCAGGTGTACACACTCAGGTGTACACACTCAGGTGTACACG |
| pLacZi-pSmCYP76AK3-F6 | AATTCGTAGCCACTTGCATCTAGCCACTTGCATCTAGCCACTTGCATCACC |
| pLacZi-pSmCYP76AK3-R6 | TCGAGGTGATGCAAGTGGCTAGATGCAAGTGGCTAGATGCAAGTGGCTACG |
| pLacZi-pSmbHLH65F | AATTCGTTCATTGACCAAATTTCATTGACCAAATTTCATTGACCAAATACC |
| pLacZi-pSmbHLH65R | TCGAGGTATTTGGTCAATGAAATTTGGTCAATGAAATTTGGTCAATGAACG |
|  | **Pull-down/EMSA** |
| pET32a-SmbHLH65F | GCCATGGCTGATATCGGATCCATGCAGCAAGAAAACAGTGGC |
| pET32a-SmbHLH65R | TGCGGCCGCAAGCTTGTCGACAAACGACCTGTAGTGATCAAG |
| pGEX4T-1-SmbHLH85F | GATCTGGTTCCGCGTGGATCCATGTTGGCTATTTCTCCTCAAATG |
| pGEX4T-1-SmbHLH85R | ACCCGGGAATTCCGGGGATCCCATCAAAGGCCAAACTTTCTC |
|  | **EMSA** |
| pGEX4T-1-SmbHLH65F | GATCTGGTTCCGCGTGGATCCATGCAGCAAGAAAACAGTGGC |
| pGEX4T-1-SmbHLH65R | ACCCGGGAATTCCGGGGATCCAAACGACCTGTAGTGATCAAG |
| pGEX4T-1-SmWRKY32-F | GATCTGGTTCCGCGTGGATCCATGGAGGATGATCGAAGAATAGTAGA |
| **Primer Name** | **Primer sequence (5'-3')** |
| pGEX4T-1-SmWRKY32-R | ACCCGGGAATTCCGGGGATCCGAAGGAAGAGTAAATTTGCATCTG |
| Bio-pSmDXS2-F | AAAGGCCATGTGTTTTTAAAAGGCCATGTGTTTTTAAAAGGCCATGTGTTTTTA |
| Bio-pSmDXS2-R | TAAAAACACATGGCCTTTTAAAAACACATGGCCTTTTAAAAACACATGGCCTTT |
| Cold-pSmDXS2-F | AAAGGCCATGTGTTTTTAAAAGGCCATGTGTTTTTAAAAGGCCATGTGTTTTTA |
| Cold-pSmDXS2-R | TAAAAACACATGGCCTTTTAAAAACACATGGCCTTTTAAAAACACATGGCCTTT |
| Cold-pSmDXS2-M-F | AAAGGCAAAAAATTTTTAAAAGGCAAAAAATTTTTAAAAGGCAAAAAATTTTTA |
| Cold-pSmDXS2-M-R | TAAAAATTTTTTGCCTTTTAAAAATTTTTTGCCTTTTAAAAATTTTTTGCCTTT |
| Bio-pSmCPS1-F | CATTTCCACATGCAATTACATTTCCACATGCAATTACATTTCCACATGCAATTA |
| Bio-pSmCPS1-R | TAATTGCATGTGGAAATGTAATTGCATGTGGAAATGTAATTGCATGTGGAAATG |
| Cold-pSmCPS1-F | CATTTCCACATGCAATTACATTTCCACATGCAATTACATTTCCACATGCAATTA |
| Cold-pSmCPS1-R | TAATTGCATGTGGAAATGTAATTGCATGTGGAAATGTAATTGCATGTGGAAATG |
| Cold-pSmCPS1-M-F | CATTTCAAAAAACAATTACATTTCAAAAAACAATTACATTTCAAAAAACAATTA |
| Cold-pSmCPS1-M-R | TAATTGTTTTTTGAAATGTAATTGTTTTTTGAAATGTAATTGTTTTTTGAAATG |
| Bio-pSmbHLH65-F | TTCATTGACCAAATTTCATTGACCAAATTTCATTGACCAAAT |
| Bio-pSmbHLH65-R | ATTTGGTCAATGAAATTTGGTCAATGAAATTTGGTCAATGAA |
| Cold-pSmbHLH65-F | TTCATTGACCAAATTTCATTGACCAAATTTCATTGACCAAAT |
| Cold-pSmbHLH65-R | ATTTGGTCAATGAAATTTGGTCAATGAAATTTGGTCAATGAA |
| Cold-pSmbHLH65-M-F | TTCAAAAAAAAAATTTCAAAAAAAAAATTTCAAAAAAAAAAT |
| Cold-pSmbHLH65-M-R | ATTTTTTTTTTGAAATTTTTTTTTTGAAATTTTTTTTTTGAA |
|  | **Subcellular localization /Dual-LUC** |
| pCsGFPBT-SmbHLH65F | TACGAACGATAGCCATGGtgATGCAGCAAGAAAACAGTGGC |
| pCsGFPBT-SmbHLH65R | CGCCCTTGCTCACCATGGcAAACGACCTGTAGTGATCAAG |
| pCsGFPBT-SmbHLH85F | TACGAACGATAGCCATGGtgATGTTGGCTATTTCTCCTCAAATG |
| pCsGFPBT-SmbHLH85R | CGCCCTTGCTCACCATGGcCATCAAAGGCCAAACTTTCTC |
|  | **Dual-LUC** |
| pCsGFPBT-SmWRKY32-F | TACGAACGATAGCCATGGtgATGGAGGATGATCGAAGAATAGTAGA |
| pCsGFPBT-SmWRKY32-R | CGCCCTTGCTCACCATGGcGAAGGAAGAGTAAATTTGCATCTG |
| pGreenⅡ-0800-luc-pSmDXS2F | gtaccgggccccccctcgaggtcgacCTGTTCGAAAGTCAAGCTTCGA |
| pGreenⅡ-0800-luc-pSmDXS2R | ggctgcaggaattcgatatcaagcttCTCTGTGTATCTCACTCTCGGCC |
| pGreenⅡ-0800-luc-pSmCPS1F | gtaccgggccccccctcgaggtcgacTGTTTTTTTTTTTCTTGATTGTGAC |
| pGreenⅡ-0800-luc-pSmCPS1R | ggctgcaggaattcgatatcaagcttTCAAATTTCCCTTTGAGTGGAGATG |
| pGreenⅡ-0800-luc-pSmbHLH65F | gtaccgggccccccctcgaggtcgacAAAGCATACGGTTACTCAAGTTCCT |
| pGreenⅡ-0800-luc-pSmbHLH65R | ggctgcaggaattcgatatcaagcttAAAATCGTTACAAAATAATTGCCCT |
